# Adenoid cystic carcinoma of the breast – an aggressive presentation with pulmonary, kidney, and brain metastases: a case report

**DOI:** 10.1186/s13256-017-1459-0

**Published:** 2017-10-29

**Authors:** Hasnae Alaoui Mhamdi, Hampig Raphael Kourie, Christiane Jungels, Philippe Aftimos, Rhizlane Belbaraka, Martine Piccart-Gebhart

**Affiliations:** 1 Department of Medical Oncology, University Hospital of Marrakech, Marrakech, Morocco; 2 Department of Oncology, Faculty of Medicine, Saint Joseph University, Beirut, Lebanon; 3 Department of Medical Oncology, Jules Bordet Institute, Université Libre de Bruxelles, Brussels, Belgium

**Keywords:** Breast, Metastatic adenoid cystic carcinoma, Triple negative, Treatment

## Abstract

**Background:**

Adenoid cystic carcinoma of the breast is a rare malignant neoplasm associated with an excellent prognosis and a very rare occurrence of metastases.

**Case presentation:**

We report the case of an aggressive presentation in a 65-year-old woman, of Belgian origin, who was diagnosed as having adenoid cystic carcinoma of the breast and developed metastases to her lung, kidney, and brain.

**Conclusions:**

We describe similar cases reported in the literature and discuss the molecular characteristics and treatment paradigm of this controversially aggressive disease entity.

## Background

More than 90% of breast cancers are ductal and lobular carcinomas; the remaining 10% represent a heterogenous group of rare histologies of breast cancer that have different clinical, pathological, and prognostic features. The major issue in the management of these rare tumors is the absence of clinical trials enrolling these patients to determine the best treatment. Adenoid cystic carcinoma (ACC) is one of these rare tumors, representing less than 0.1% of all patients diagnosed with breast cancer. It affects mainly women aged 50 to 60. It is usually a “triple negative” breast cancer expressing neither hormone receptors nor HER2-neu [1, 2]. Despite being a triple negative breast cancer (TNBC), ACC is usually an indolent malignancy with a very good prognosis, when confined to the breast. Lymph node involvement is very rare not exceeding 2%, and distant metastases are exceptional [3, 4].

Here, we report a rare case of ACC of the breast metastasizing to the kidney and the brain. We present a complete review of the literature on similar cases and we discuss the corresponding molecular characteristics and treatment modalities of this intriguing entity.

## Case presentation

A 65-year-old woman, of Belgian origin, with an unremarkable past medical history and no family history, was diagnosed in 2009 with ACC of her left breast. The tumor measured 8 cm (T3) and was triple negative. She underwent mastectomy with lymph node dissection, followed by radiation therapy. All lymph nodes were free of disease.

The disease relapsed first locally as a regular, mobile, and dense nodule of 12.7 × 11.7 mm; it was resected in December 2013. Then the disease relapsed as a pulmonary nodule in 2014, histologically confirmed, treated first with eight cycles of mitoxantrone with good tolerance followed by pulmonary lobectomy in view of the persistence of lesions at the lower lobe of her left lung. A next generation sequencing of 400 genes of the primary tumor did not show any significant gene aberration.

In 2015, a kidney cyst was detected; a fine-needle aspiration of this cyst showed malignant cells. A left nephrectomy confirmed a distant metastasis of the ACC of the breast.

In 2016, neurologic symptoms prompted a brain magnetic resonance imaging (MRI), which revealed metastasis associated with severe edema. Following surgical resection, the pathology examination confirmed a distant metastasis of ACC of the breast. She was treated with complementary whole brain radiation therapy and no complications were reported. The disease rapidly progressed after 3 months which correlated with the appearance of pancreatic metastases. She was included in a clinical trial.

## Discussion

Adenoid cystic breast carcinoma is a rare form of breast cancer, which is named after its microscopic appearance [5, 6]. It is a non-aggressive type of breast carcinoma with a very good chance of full recovery and it has a low propensity for metastasis [7, 8].

Only two cases of ACC with brain metastases and two other cases with kidney metastases were reported in the literature [9–12]; the characteristics of the patients, their tumors, and their management are summarized in Table 1. To the best of our knowledge, this is the first case of ACC that metastasized to lung, kidney, and brain.

**Table 1:** Literature review of other reported cases of advanced cystic adenoid carcinoma of the breast

Cases	Patient (age (years)/gender)	Distant metastasis	Treatment	Survival/prognostic	Time to relapse
Herzberg *et al*. [9]	57, female	Kidney	Mastectomy/nephrectomy	5-year disease-free survival, then lost to follow-up	Distant relapse: 6 years
Vranic *et al*. [10]	76, female	Kidney	Mastectomy/radical nephrectomy	No information after relapse	Distant relapse: 5 years
Koller *et al*. [11]	49, female	Brain, lungs	Breast surgery/Chemotherapy/radiation/left parieto-occipital craniotomy	No relapse at 20 months from the surgery	Local relapse: 1 year. Distant relapse: 8 years
Silva *et al*. [12]	37, female	Brain, liver, lungs, bone	Radical mastectomy/adjuvant chemotherapy (six cycles of adriamycin and cyclophosphamide)/radiotherapy/palliative chemotherapy (docetaxel plus vinorelbine and 5FU in second line)	The patient died 1 year after developing brain metastasis	Distant relapse: 2 years
Our case	65, female	Brain, lung, kidney	Mastectomy/palliative chemotherapy (mitoxantrone)/thoracotomy/radical nephrectomy/craniotomy plus radiotherapy	Patient still alive, now presenting liver lesions	Local relapse: 4 years. Distant relapses: 5 years

*5FU* 5-fluorouracil

The initial treatment of our patient was in line with published guidelines. Local therapy can consist in a simple or modified radical mastectomy or lumpectomy associated with radiotherapy [13, 14]. Axillary node dissection is not recommended in view of the very low incidence of axillary lymph node metastasis that does not exceed 2% [8]. Similarly, there is no indication for adjuvant chemotherapy and several small studies did not suggest a survival benefit on overall survival, when adding adjuvant chemotherapy [15].

Due to the rarity of ACC with metastases, treatment recommendations are sparse. Some physicians are extrapolating the treatment of ACC of the breast from the one of the salivary glands, the most frequent localization of ACC. For patients with metastatic disease, some cytotoxic agents have been used including vinorelbine, 5-fluorouracil (5FU), and doxorubicin [11, 12].

Mitoxantrone was evaluated in advanced ACC of the head and neck region with no significant anti-tumor activity in one trial and modest activity in another (12% of response rate) [16, 17]. This drug was not tested in ACC of the breast before. We administered mitoxantrone to our patient for eight cycles and she presented a stable disease in her lung metastases. Mitoxantrone was chosen because of its favorable toxicity profile, including no hair loss.

We did evaluate the genetic alterations of her tumor hoping for actionable genetic aberrations. Unfortunately, our target gene sequencing did not reveal any mutation or copy number aberration that would justify the use of a particular targeted drug. A recently published study described specific genetic alterations in rare breast cancer tumors (micropapillary, metaplastic, pleomorphic lobular) [14]. In another reported case in the literature of ACC metastasizing to the kidney, a PI3K mutation was detected [10]. Progress in these rare tumor types might occur through the conduct of basket trials in the future.

## Conclusions

ACC is a rare neoplasm of the breast. Management of a metastatic situation presents challenges for medical oncologists. The benefit of using chemotherapy and the definition of which agent should be used remain unknown because of the paucity of reported cases in metastatic situations. It is necessary to have other cases before establishing a consensus for this type of tumor.

## Abbreviations

5FU: 5-Fluorouracil
ACC: Adenoid cystic carcinoma
TNBC: Triple negative breast cancer
